# Causal effect of severe and non‐severe malaria on dyslipidemia in African Ancestry individuals: A Mendelian randomization study

**DOI:** 10.1111/ahg.12555

**Published:** 2024-03-15

**Authors:** Mariam Traore, Harouna Sangare, Oudou Diabate, Abdoulaye Diawara, Cheickna Cissé, Oyekanmi Nashiru, Jian Li, Jeffrey Shaffer, Mamadou Wélé, Seydou Doumbia, Tinashe Chikowore, Opeyemi Soremekun, Segun Fatumo

**Affiliations:** ^1^ The African Computational Genomics (TACG) Research Group Medical Research Council /Uganda Virus Research Institute and London School of Hygiene and Tropical Medicine Uganda Research Unit Entebbe Uganda; ^2^ African Center of Excellence in Bioinformatics University of Sciences, Techniques and Technologies of Bamako Bamako Mali; ^3^ Department of Mathematics and Informatics, Faculty of Sciences and Techniques (FST) University of Sciences, Techniques and Technologies of Bamako (USTTB) Bamako Mali; ^4^ Faculty of Medicine and Odonto‐stomatology University of Sciences, Techniques and Technologies of Bamako Bamako Mali; ^5^ H3Africa Bioinformatics Network (H3ABioNet) Node, Centre for Genomics Research and Innovation NABDA/FMST Abuja Nigeria; ^6^ School of Public Health and Tropical Medicine Tulane University New Orleans USA; ^7^ Department of Biological Sciences, Faculty of Sciences and Techniques University of Sciences, Techniques and Technologies of Bamako Bamako Mali; ^8^ MRC/Wits Developmental Pathways for Health Research Unit, Department of Pediatrics, Faculty of Health Sciences University of the Witwatersrand Johannesburg South Africa; ^9^ Sydney Brenner Institute for Molecular Bioscience, Faculty of Health Sciences University of the Witwatersrand Johannesburg South Africa; ^10^ Molecular Bio‐computation and Drug Design Laboratory, School of Health Sciences University of KwaZulu‐Natal Durban South Africa; ^11^ Department of Non‐communicable Disease Epidemiology (NCDE) London School of Hygiene and Tropical Medicine London UK

**Keywords:** causal effect, dyslipidemia, Malaria, Mendelian randomization

## Abstract

**Background:**

Dyslipidemia is becoming prevalent in Africa, where malaria is endemic. Observational studies have documented the long‐term protective effect of malaria on dyslipidemia; however, these study designs are prone to confounding. Therefore, we used Mendelian randomization (MR, a method robust to confounders and reverse causation) to determine the causal effect of severe malaria (SM) and the recurrence of non‐severe malaria (RNM) on lipid traits.

**Method:**

We performed two‐sample MR using genome wide association study (GWAS) summary statistics for recurrent non‐severe malaria (RNM) from a Benin cohort (*N* = 775) and severe malaria from the MalariaGEN dataset (*N* = 17,000) and lipid traits from summary‐level data of a meta‐analyzed African lipid GWAS (MALG, *N* = 24,215) from the African Partnership for Chronic Disease Research (APCDR) (*N* = 13,612) and the Africa Wits‐IN‐DEPTH partnership for genomics studies (AWI‐Gen) dataset (*N* = 10,603).

**Result:**

No evidence of significant causal association was obtained between RNM and high‐density lipoprotein cholesterol (HDL‐C), low‐density lipoprotein cholesterol (LDL‐C), total cholesterol and triglycerides. However, a notable association emerged between severe malarial anaemia (SMA) which is a subtype of severe malaria and reduced HDL‐C levels, suggesting a potential subtype‐specific effect. Nonetheless, we strongly believe that the small sample size likely affects our estimates, warranting cautious interpretation of these results.

**Conclusion:**

Our findings challenge the hypothesis of a broad causal relationship between malaria (both severe and recurrent non‐severe forms) and dyslipidemia. The isolated association with SMA highlights an intriguing area for future research. However, we believe that conducting larger studies to investigate the connection between malaria and dyslipidemia in Africa will enhance our ability to better address the burden posed by both diseases.

## 1 INTRODUCTION

Malaria remains one of the world's challenging infectious diseases despite the recent progress in reducing its burden. In 2021, an estimated 247 million cases and 619,000 deaths related to malaria were reported according to the World Health Organization (2022). Africa accounts for above 90% of malaria cases and deaths (Monroe et al., 2022). This alarming statistic underscores the persistent and pervasive nature of malaria, primarily caused by protozoan parasites of the genus *Plasmodium* and transmitted by female *Anopheles* mosquitoes (Monroe et al., 2022).

Simultaneously, dyslipidemia which is characterized by abnormal levels of one or more of the following lipid traits: low‐density lipoproteins (LDL‐C), high‐density lipoproteins (HDL‐C), total cholesterol (TC) and triglycerides (TG) is emerging as a critical public health concern (Pirillo et al., 2021; Sarfo, 2018). Dyslipidemia is one of the main risk factors for atherosclerosis and cerebrovascular disease (Gao et al., 2021). In fact, the presence of increased blood lipid levels exacerbates atherosclerosis, which is considered the prime risk factor for strokes, peripheral vascular disease and heart diseases (Milad et al., 2017). Alarmingly, the prevalence of dyslipidemia is on the rise in the African adult population (Gebreegziabiher et al., 2021). In a meta‐analysis including 177 studies on the prevalence of dyslipidemia among adults in Africa, a prevalence of 25,5% for an elevated level of total cholesterol, 19,5% for low levels of HDL cholesterol, 21,4% for a high level of LDL,17% for elevated levels of triglycerides (Noubiap et al., 2018). A number of studies have evaluated the role of dyslipidemia on the severity of malaria, but none have assessed the reverse impact of malaria on dyslipidemia. Considering the rise of dyslipidemia in Sub‐Saharan Africa and the effect malaria has on the body's metabolism, the causal association of malaria with blood lipid levels needs further investigation.

**FIGURE 1:**
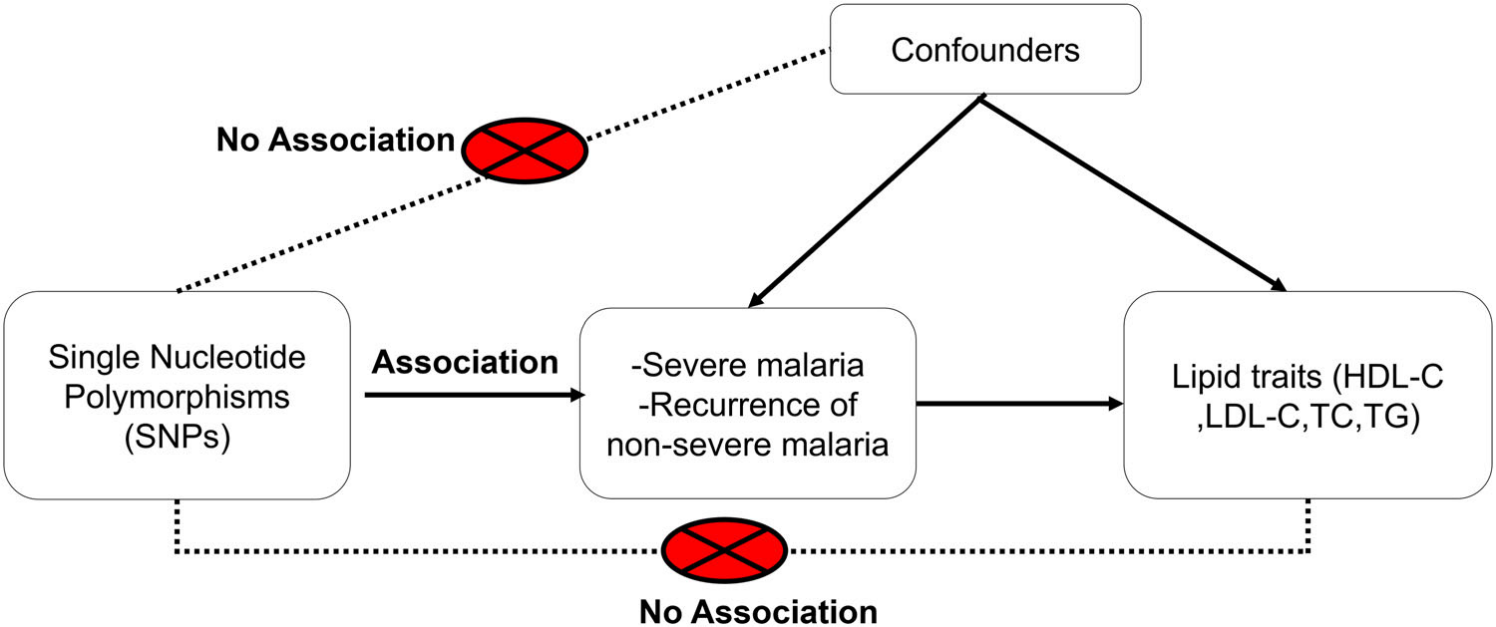
Mendelian randomization study design. HDL, High‐density lipoprotein; LDL, low‐density lipoprotein; TC, total cholesterol; TG, triglycerides.

Mendelian randomization (MR) is an analytical method that utilizes genetic variations established at conception as instruments for estimating the causal effects of modifiable risk factors influencing population health. (Davey Smith & Hemani, 2014; Davies et al., 2018; Smith & Ebrahim, 2003). Similar to a randomized control trial, MR utilizes genetic randomization, where individuals are grouped based on their genetic variants determined before birth. This approach assumes that individuals with specific genetic variants will experience the exposure differently from those without, leading to divergent outcomes. Using genetic variants as instruments helps mitigate confounding, addressing a major challenge in observational studies. (Burgess & Thompson, 2015; Davies et al., 2018). MR has been used to study the causal relationship between malaria and other diseases, such as hypertension (Etyang et al., 2019). Other MR to studies have assessed the relationship between lipid traits and other health problems, such as kidney function (Rasheed et al., 2021), coronary artery disease (Burgess & Harshfield, 2016) and breast cancer (Johnson et al., 2020). However, no study has been undertaken to determine the causal relationship between malaria and lipid traits. In this study, we bridge this knowledge gap by conducting a two‐sample MR using data from genome‐wide association studies (GWAS) to explore the effects of both severe and non‐severe malaria on dyslipidemia (study design in Figure 1). Our approach is unique in its application of MR to this context, potentially providing novel insights into the complex interactions between infectious diseases and metabolic health, with a specific focus on a population heavily burdened by both malaria and rising dyslipidemia rates.

## 2 DATA AND METHODS

### 2.1 Data source

#### 2.1.1 Recurrent non‐severe malaria

The malaria exposure data was obtained from a GWAS study of non‐severe malaria conducted in Benin, two cohorts of 525 and 250 infants used as discovery and replication cohorts, respectively (Milet et al., 2019). The discovery cohort was composed of 525 infants who were followed up from birth until 18 months in a study conducted by Le Port et al., in nine villages of the Tori‐Bossito district located 40 km North‐East of Cotonou from June 2007 to January 2010 (Le Port et al., 2012). For the 525 infants included in the discovery sample, the mean (SD) follow‐up duration was 16.9 months (2.83). Notably, 342 infants (65.1%) experienced at least one mild malaria attack, with the episodes ranging from one to 10. In the GWAS study (Milet et al., 2019), recurrence of mild malaria attacks (RMM) and recurrence of malaria infections (RMI) were the outcomes of the association analysis of the GWAS study. The definition of a mild malaria attack was a positive rapid diagnosis test (RDT), or thick blood smear microscopy (TBS) coupled with fever (axillary temperature ≥ 37.5°C) or the presence of fever during the preceding 24 h (Milet et al., 2019).

#### 2.1.2 Severe malaria

Summary statistics for severe malaria and its sub‐phenotypes were extracted from a large GWAS cohort study of 17,000 severe malaria cases and population controls from 11 countries, including nine African countries (Band et al., 2019). We downloaded the per‐population summary statistics for each African country and performed a meta‐analysis on them using the software METAL (Willer et al., 2010). The meta‐analysis was conducted for the overall severe malaria and for the sub‐phenotypes of severe malaria reported in the study: cerebral malaria (CM), severe malarial anaemia (SMA) and ‘other’ subtypes of severe malaria (Band et al., 2019). After meta‐analysis, we then used all SNPs that had a genome wide association (p < 5 × 10^−8^). This threshold was carefully chosen to ensure that only the most statistically robust and biologically relevant genetic associations were included in our analysis, thereby enhancing the reliability and validity of our findings.

### 2.2 Outcome data

Genetic instruments for lipid traits were derived from summary statistics of the MALG cohort (*N* = 24,215), which is a meta‐analysis of the summary statistics from AWI‐Gen study (the Africa Wits‐INDEPTH partnership for Genomics studies) (Choudhury et al., 2022) with summary statistics from four African cohorts featured in the Gurdasani et al., 2019 study (2019): the Uganda Genome Resource (UGR) study, the Africa‐America Diabetes Mellitus (AADM) study, the Durban Diabetes Study (DDS) and the Durban Case Control (DCC) study. The analysis focused on SNPs present in the final AWI‐Gen dataset and at least three of the other four cohorts, utilizing the Han and Eskin's random effects model (Choudhury et al., 2022).

### 2.3 Genetics instruments

We used SNPs which were found to be associated at *p*‐value < 5 × 10^−8^ with the recurrence of mild malaria attacks (RMM) (Milet et al., 2019) and with severe malaria and its sub‐phenotypes (Band et al., 2019). These SNPs were clumped at linkage disequilibrium (LD) *r* ^2^ < 0.001 using the 1000 Genomes African reference panel in a 500 kb window. After, the exposure and outcome data were harmonized to ensure that an SNP's effect on the exposure and outcome corresponds to the same allele. We excluded palindromic SNPs with a minor allele frequency > 40%. The SNPs (see Supporting Information) that remained after all these steps were finally used as instruments for each respective outcome.

### 2.4 Mendelian randomization analysis

The MR analysis was performed using the random‐effects inverse‐variance weighted method. In this approach, a regression of the genetic associations with the outcome on the genetic associations with exposure is performed (Burgess et al., 2019). The Wald ratio method was used to perform the MR analysis for the recurrent non‐severe malaria and the subtype SMA because only one SNP was used as instrument (IV) for those traits. A sensitivity analysis was run to increase confidence in the validity of the results and to limit the bias of pleiotropic effects. As pleiotropy effects are commonly and inevitably encountered in this type of analysis, this step was crucial to enhance the robustness of the findings. This sensitivity analysis was done with robust methods: MR‐Egger and median weighted methods (Burgess et al., 2019). Mendelian randomization‐Egger (MR‐Egger) is an analysis method which consists of testing for directional pleiotropy as well as providing a consistent estimate of the causal effect in the absence of violation of the InSIDE (Instrument Strength Independent of Direct Effect) rule even if all variants are invalid (Boehm & Zhou, 2022). The median‐based method can provide a consistent estimate of the causal effect even if only half of the genetic variants are valid (Burgess et al., 2019). In our study, the significant statistical level was 0.05. All statistical analyses were conducted using R (version 4.0.1, The R Foundation for Statistical Computing Platform, Vienna, Austria) with the R packages ‘TwoSampleMR’ (Hemani et al., 2018) and ‘MedelianRandomization’ (Yavorska & Burgess, 2017).

### 2.5 Ethical statement

Since all data used in this study were sourced from publicly available summary‐level GWAS datasets and did not involve the use of individual‐level data, there was no need for additional ethical approval or patient consent.

## 3 RESULTS

### 3.1 Participants characteristics

The characteristics of the participants for both malaria and lipid traits are listed in Table 1. The proportion of male participants in the data on recurrent non‐severe malaria is 49.7.

**TABLE 1:** Characteristics of the cohorts of the exposure and the outcome.

Characteristics	Severe malaria	Recurrent non‐severe malaria	Lipid traits
Sample size	15,088	525	24,215
Gender male n (%)	NA	261(49.7)	NA

*Note*: NA indicates not applicable.

### 3.2 Recurrence of mild malaria attack against lipids traits

**FIGURE 2:**
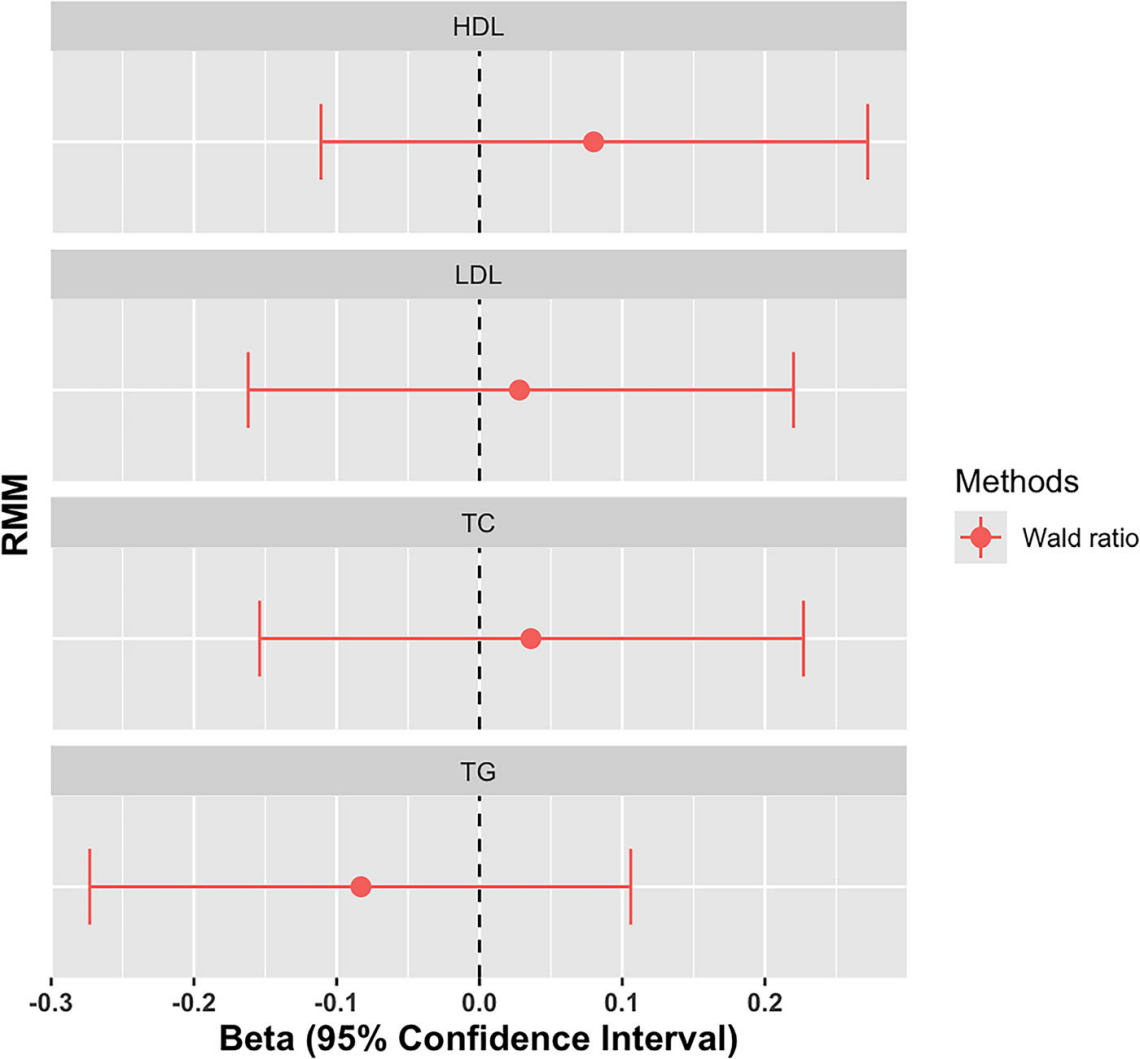
Forest plot showing the beta estimates with their 95% confidence intervals of the Mendelian randomization (MR) analysis of the effect of recurrent mild malaria (RMM) on lipid traits. The beta estimates are represented in dots and their respective confidence interval in lines. HDL, High‐density lipoprotein; LDL, low‐density lipoprotein; TC, total cholesterol; TG, triglycerides.

In the main analysis (IVW) (Table 2), There was no evidence of a statistically significant causal association between the recurrence of mild malaria attack and the level of HDL‐C (0.080 (−0.111 to 0.272), *p*‐value = 0.410), LDL‐C level (0.028 (−0.162 to 0.220), *p*‐value = 0.768) total cholesterol (0.036 (−0.154 to 0.227), *p*‐value = 0.705) and triglycerides (−0.083 (−0.273 to 0.106), *p*‐value = 0.390). The forest plot illustrating the MR results is shown in Figure 2.

**TABLE 2:** Summary of MR analysis of the causal effect of recurrent mild malaria (RMM) on lipid traits.

Traits	Method	NSNP	Beta (95%CI)	SE	*p*‐Value
RMM vs HDL	Wald ratio	1	0.080 (−0.111 to 0.272)	0.097	0.410
RMM vs LDL	Wald ratio	1	0.028 (−0.162 to 0.220)	0.097	0.768
RMM vs TC	Wald ratio	1	0.036 (−0.154 to 0.227)	0.097	0.705
RMM vs TG	Wald ratio	1	−0.083 (−0.273 to 0.106)	0.097	0.390

Abbreviations: CI, confidence Interval; HDL, high‐density lipoprotein; LDL, low‐density lipoprotein; NSNP, number of SNP (single‐nucleotide polymorphism); SE, standard error; TC, total cholesterol; TG, triglycerides.

### 3.3 Effect of the overall severe malaria on lipid traits

**FIGURE 3:**
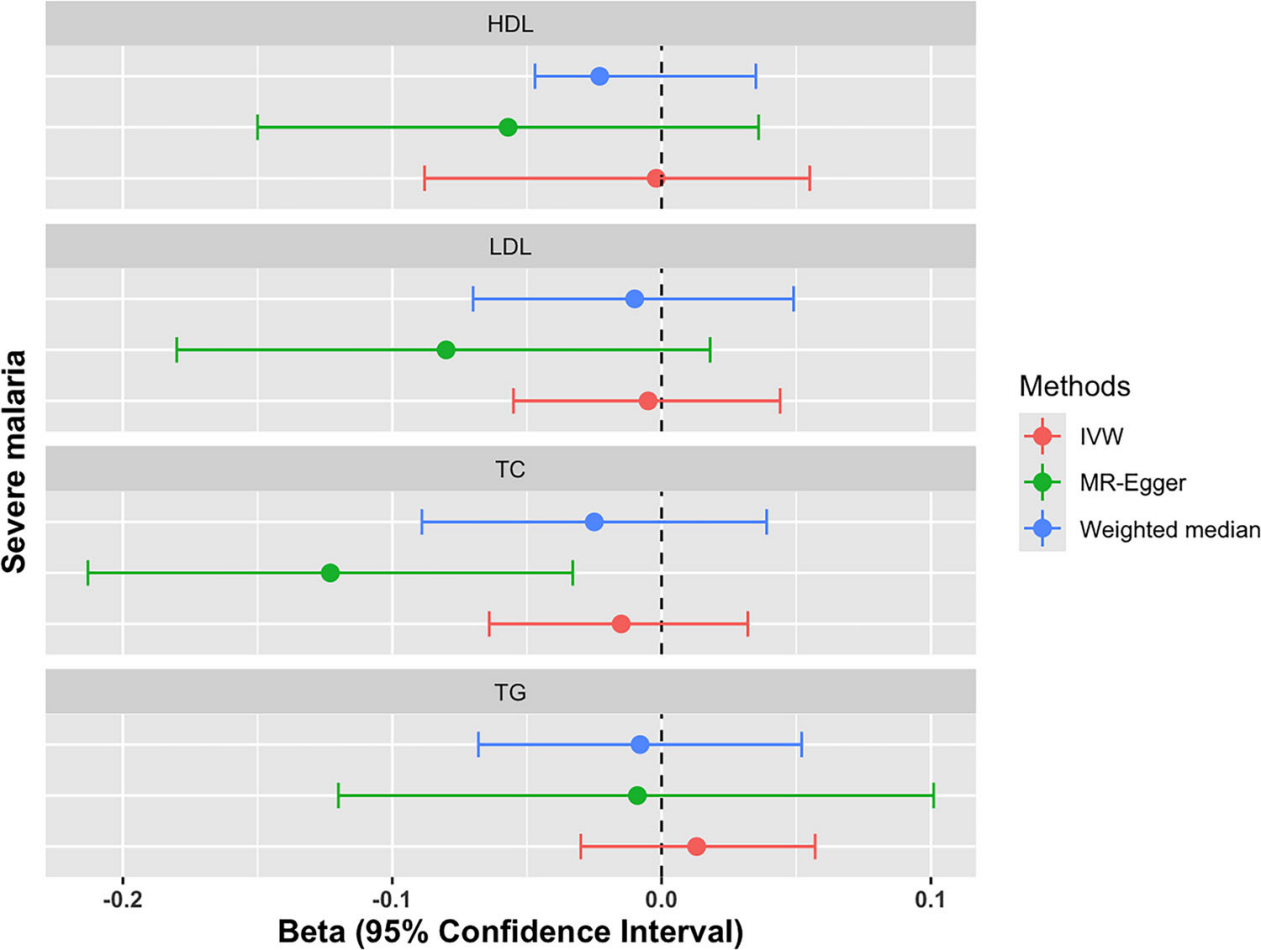
Forest plot showing the beta estimates with their 95% confidence intervals of the Mendelian randomization (MR) analysis of the effect of the overall severe malaria on lipid traits. The beta estimates are represented in dots and their respective confidence interval in lines. The red line shows the result obtained with IVW method, the green line the result obtained with MR‐Egger and the blue line the result obtained with the weighted median method. HDL, High‐density lipoprotein; LDL, low‐density lipoprotein; TC, total cholesterol; TG, triglycerides.

As shown in the (Table 3) we did not find any significant evidence of association between severe malaria and increase levels of HDL‐C (IVW Beta: −0.002 95% CI: −0.088 to 0.055; *p*‐value = 0.923), LDL‐C (IVW Beta: −0.005 95% CI: −0.055 to 0.044; *p*‐value = 0.825), total cholesterol (IVW Beta: −0.015 95% CI: −0.064 to 0.032; *p*‐value = 0.522) and triglycerides (IVW Beta: 0.013 95% CI: −0.030 to 0.057; *p*‐value = 0.545). However, a significant association was observed between severe malaria and total cholesterol with the MR‐egger method (IVW Beta: −0.123 95% CI: −0.213 to −0.033; *p*‐value = 0.020). The estimates and their confidence intervals are represented as a forest plot in the Figure 3.

**TABLE 3:** Summary of MR analysis of the causal effect of severe malaria on lipid traits.

Traits	Methods	NSNP	Beta (95%CI)	SE	*P*‐value
SM vs HDL	IVW	13	−0.002 (−0.088 to 0.055)	0.023	0.923
	MR‐Egger	13	−0.057(−0.150 to 0.036)	0.047	0.257
	Weighted Median	13	−0.023 (−0.047 to 0.035)	0.030	0.441
SM vs LDL	IVW	13	−0.005 (−0.055 to 0.044)	0.025	0.825
	MR‐Egger	13	−0.080 (−0.180 to 0.018)	0.050	0.140
	Weighted Median	13	−0.010 (−0.070 to 0.049)	0.030	0.732
SM vs TC	IVW	13	−0.015 (−0.064 to 0.032)	0.024	0.522
	MR‐Egger	13	−0.123 (−0.213 to −0.033)	0.045	0.020
	Weighted Median	13	−0.025 (−0.089 to 0.039)	0.032	0.446
SM vs TG	IVW	13	0.013 (−0.030 to 0.057)	0.022	0.545
	MR‐Egger	13	−0.009 (−0.120 to 0.101)	0.056	0.794
	Weighted Median	13	‐0.008 (−0.068 to 0.052)	0.030	0.871

Abbreviations: CI, Confidence Interval. HDL, High‐density lipoprotein; IVW, inverse variance weighted; LDL, low‐density lipoprotein; MR‐Egger, Mendelian randomization‐Egger; NSNP, number of SNP (single‐nucleotide polymorphism); OR, ODD RATIO; SE, standard error; SM, severe malaria; TC, total cholesterol; TG, triglycerides.

### 3.4 Effect of the sub phenotypes of severe malaria on lipid traits

**FIGURE 4:**
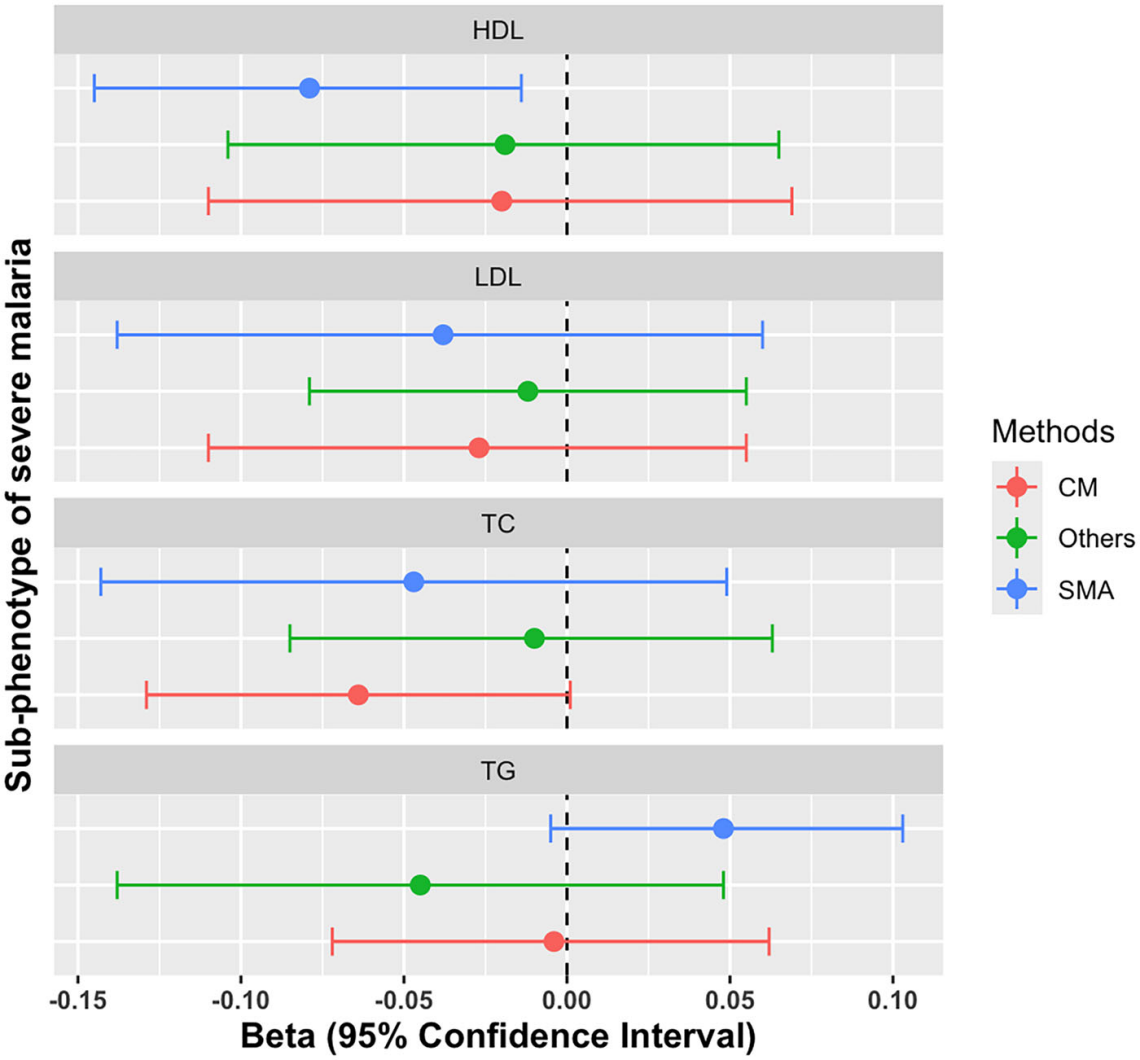
Forest plot showing beta estimates with their 95% confidence intervals of the MR analysis of the effect of the sub phenotypes of severe malaria on lipid traits. The beta estimates are represented in dots and their respective confidence interval in lines. The red line shows the result obtained with Cerebral malaria (CM), the green line the result obtained with other subtypes and the blue line the result obtained with severe malarial anaemia (SMA). HDL, High‐density lipoprotein; LDL, Low‐density Lipoprotein; TC, Total cholesterol; TG, Triglycerides.

The MR analysis between different subtypes of severe malaria (Table 4) suggests severe malarial anaemia (SMA) to be associated with the risk of having low levels of HDL‐C (Wald ratio method, Beta: −0.079, 95% CI: −0.145 to −0.014, *p*‐value = 0.017.) We did not find any significant evidence of causal association between the others subtypes and lipid traits. The estimates and their confidence intervals are represented as a forest plot in the Figure 4.

**TABLE 4:** Summary of MR analysis of the causal effect of different subtypes of severe malaria on Lipid Traits.

Traits	Sub‐phenotypes of SM	NSNP	Beta (95%CI)	SE	*p*‐Value
HDL	CM	2	−0.020 (−0.110 to 0.069)	0.045	0.659
	SMA	1	−0.079 (−0.145 to −0.014)	0.033	0.017
	Other	3	−0.019 (−0.104 to 0.065)	0.043	0.653
LDL	CM	2	−0.027 (−0.11 to 0.055)	0.042	0.511
	SMA	1	−0.038 (−0.138 to 0.060)	0.050	0.442
	Other	3	−0.012 (−0.079 to 0.055)	0.034	0.725
TC	CM	2	−0.064 (−0.129 to 0.001)	0.033	0.055
	SMA	1	−0.047 (−0.143 to 0.049)	0.049	0.337
	Other	3	−0.010 (−0.085 to 0.063)	0.037	0.773
TG	CM	2	−0.004 (−0.072 to 0.062)	0.034	0.887
	SMA	1	0.048 (−0.005 to 0.103)	0.027	0.076
	Other	3	−0.045 (−0.138 to 0.048)	0.047	0.340

Abbreviations: CI, confidence interval; CM: cerebral malaria; HDL: high‐density lipoprotein; IVW: inverse variance weighted; LDL: low‐density lipoprotein; MR‐Egger: Mendelian randomization‐Egger; NSNP, number of SNP (single‐nucleotide polymorphism); OR, odd ratio; Other: ‘other’ subtypes of severe malaria; SM, severe malaria; SMA, severe malarial anaemia; SE, standard error; TC: total cholesterol; TG: triglycerides.

## 4 DISCUSSION

This two sample MR study investigated the causal effect of severe malaria and recurrent non‐severe malaria on the level of high‐density lipoprotein cholesterol, low‐density lipoprotein cholesterol, total cholesterol and triglycerides using summary statistics of malaria and lipid traits. We did not find significant causal association between recurrent non‐severe malaria as well as severe malaria and the levels of HDL‐C, LDL‐C total cholesterol and triglycerides. Using the MR‐Egger method especially with severe malaria allowed us to ensure that our results were not affected by horizontal pleiotropy (Intercept = −0.0175, *p* = 0.0119) for HDL‐C, (Intercept = 0.0123, *p* = 0.203) for LDL‐C, (Intercept = 0.0210, *p* = 0.026) for total cholesterol and (Intercept = −0.0020, *p* = 0.776) for triglycerides. It should be noted that the sensitivity analyses performed in this section primarily focus on assessing the potential impact of horizontal pleiotropy.

Among the instruments we used for the overall severe malaria, two SNPs (rs113850170, rs334) were associated with the *HBB* gene, two SNPs (rs8176751, rs9411466) were associated with *HBO* gene. Regarding the subtypes of SM, SNPs (rs113850170, rs334) were associated with the *HBB* gene for cerebral malaria, while one SNP (rs334) were associated with the *HBB* gene for the severe malarial aneamia and the ‘other subtype’.

To the best of our knowledge, this study represents the first foray into exploring the causal links between severe and non‐severe malaria and lipid profiles through MR providing a novel perspective on the intersection of infectious diseases and metabolic traits. While our study identifies a connection between SMA and reduced HDL‐C, it does not establish a causal relationship between severe malaria or recurrent non‐severe malaria infections and other lipid traits. However, changes in lipid profile during malaria infection has been reported in several studies. As evidence, individuals with previous episodes of malaria and living in a malaria‐endemic area had hypocholesterolemia (Neves et al., 2013). Warjri et al. reported a low LDL‐C and total cholesterol level in malaria patients compared to non‐malaria patients (2016). The lack of association between malaria and low level of HDL‐C and high level of triglycerides was surprising, given that several studies reported significant decreases in HDL‐C levels and increases in triglycerides levels in malaria patients compared to the healthy patient (no malaria) (Visser et al., 2013). Furthermore, there tend to be a decrease of HDL‐C levels and a rise in the level of triglycerides, not just during malaria, but during any systemic inflammatory response (Feingold et al., 2000; Tsoupras et al., 2018). A meta‐analysis of different studies of serum lipid profile during malaria found that 30 studies reported a significantly lowered total cholesterol level in malaria patients compared to non‐malaria patients. and 13 studies reported a reduced level of LDL‐C in malaria patients (Visser et al., 2013).

To contextualize these unexpected findings, it is crucial to delve into potential mechanisms that link malaria infection and lipid metabolism. The mechanism that results in the changes in lipid profile during malaria infection is uncertain. Still, according to some studies (Labaied et al., 2011; Visser et al., 2013), the lipid's role during the host‐parasite interaction may explain this. In fact, the liver, which is the leading site of *Plasmodium* infection, is also where lipid is synthesized (Labaied et al., 2011). Proteins and lipids such as cholesterol and lipoprotein are needed in considerable quantities in cellular development and cell division in the parasite (Labaied et al., 2011). Therefore, the parasite's reliance on host lipids and cholesterol for its development could explain observed alterations in lipid levels during infection (Visser et al., 2013).

Our study is an important addition to the literature regarding the relationship between lipid parameters and malaria because of the use of the MR method. Another strength of this study is the fact that we made sure to use strong instrumental variables. We also performed different sensitivity analysis to test the consistency of our results and to ensure that they were not affected by horizontal pleiotropy. Furthermore, we focused our analysis on African ancestry data, especially since African ancestry individuals are underrepresented in genetics studies.

Our study has some limitations. First, the sample size of one of our exposures (recurrent non‐severe malaria) data was small (525 cases and 250 control). This illustrates the need for more GWAS studies, including a larger population from Africa for malaria and other health‐related situations such as lipids traits and other cardiometabolic traits. Second, there was age‐related gap between the population (infants) of the non‐severe malaria data and the people (adults) of the outcome data; this may affect the MR results.

This two sample MR suggest a significant association between SMA, a specific subtype of severe malaria, and the risk of low HDL‐C levels. Beyond this association, we did not find meaningful long‐term changes in lipid profiles attributable to severe malaria or recurrent non‐severe malaria episodes. Nonetheless, this MR study acts as a caltalyst for future inquiries into the intricate relationship between infectious diseases, such as malaria and lipid metabolism. As we chart the course forward, larger GWAS, diverse population representation and investigations into the long‐term effects of malaria exposure emerge as pivotal components in advancing our understanding of the broader health implications.

## AUTHOR CONTRIBUTIONS

**Mariam Traore**: Data analysis; writing—original draft. **Harouna Sangare**: Supervision; writing—review and editing. **Oudou Diabate**: Writing—review and editing. **Abdoulaye Diawara**: Writing—review and editing. **Cheickna Cissé**: Writing—review and editing. **Oyekanmi Nashiru**: Writing—review and editing. **Jian Li**: Writing—original draft; writing—review and editing. **Jeffrey Shaffer**: Writing—review and editing. **Mamadou Wélé**: Writing—review and editing. **Seydou Doumbia**: Writing—review and editing. **Tinashe Chikowore**: Writing—review and editing. **Opeyemi Soremekun**: Data curation; supervision; writing—original draft; writing—review and editing. **Segun Fatumo**: Data curation; supervision; writing—review and editing. All authors have read and approved the final manuscript.

## CONFLICT OF INTEREST STATEMENT

The authors declare that there is no conflict of interest, financial or otherwise.

## Supporting information

Supplementary information

## Data Availability

The instruments data for the recurrence of non‐severe malaria has been retrieved from the paper https://link.springer.com/article/10.1007/s00439‐019‐02079‐5. The dataset for the severe malaria is available on MalariaGEN website. The lipid traits data is available on GWAS Catalog (**GCST90101745** for HDL‐C, **GCST90101746** for LDL‐C, **GCST90101747** for Total cholesterol, **GCST90101748** for Triglycerides).
